# A transcriptomic landscape analysis of human necrotizing enterocolitis: Important roles of immune infiltration

**DOI:** 10.1002/pdi3.1

**Published:** 2023-06-09

**Authors:** Zhuojun Xie, Quan Kang, Yulu Shi, Junbao Du, Hao Jiang

**Affiliations:** ^1^ Stem Cell Biology and Therapy Laboratory The Children's Hospital of Chongqing Medical University National Clinical Research Center for Child Health and Disorders Ministry of Education Key Laboratory of Child Development and Disorders Chongqing Key Laboratory of Pediatrics Chongqing China; ^2^ Department of General Surgery The Children's Hospital of Chongqing Medical University National Clinical Research Center for Child Health and Disorders Ministry of Education Key Laboratory of Child Development and Disorders Chongqing Key Laboratory of Pediatrics Chongqing China; ^3^ Molecular Oncology Laboratory The University of Chicago Medical Center Chicago Illinois USA; ^4^ Pediatric Ward 1 Taihe Hospital Shiyan City Hubei China

**Keywords:** gene expression, immune infiltration, necrotizing enterocolitis, RNA‐seq

## Abstract

Necrotizing enterocolitis (NEC) is one of the most common and destructive diseases in neonates and an unpredictable surgical emergency. However, the molecular pathological mechanism of NEC is still not well understood. This study was designed to provide a molecular basis for the pathogenesis of human NEC through bioinformatics analysis and immune infiltration. For RNA‐Seq, DEseq2 algorithm was used to identify differentially expressed genes (DEGs) and to perform functional enrichment analysis. Immune infiltration was analyzed by CIBERSORT algorithm. A total of 34,712 genes were detected and 7463 DEGs were identified in this study. Gene Ontology analysis revealed that DEGs were mainly involved in CCR1 chemokine receptor binding, transporter activity, growth factor binding, etc. KEGG pathway analysis showed that the DEGs were significantly enriched in the toll‐like receptor signaling pathway, Th17 cell differentiation, and cytokine–cytokine receptor interaction. The immune infiltration profiles varied significantly between NEC, NEC self‐control, and normal intestinal tissues. Finally, the expression levels of 21 DEGs were verified by reverse transcription quantitative real‐time PCR. Our findings may provide new insights into the development of NEC.

## Graphical abstract

In this paper, small intestinal samples from human necrotizing enterocolitis (NEC) were collected for transcriptome sequencing. Further, bioinformatics analysis of the sequenced data aimed to identify the key differentially expressed genes in the development of NEC as well as the biological functions and pathways that may play an important role. In addition, differences in immune infiltration between different tissues were compared in order to provide a broader theoretical basis for the exploration of the pathogenesis of NEC.

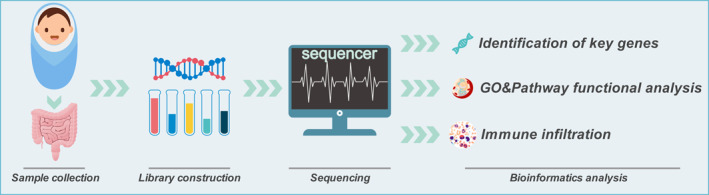

## 1 INTRODUCTION

Necrotizing enterocolitis (NEC) is a major cause of morbidity and mortality in the neonatal intensive care unit. The vast majority (>90%) of NEC occurs in preterm infants, especially those who are of very low birth weight (<1500 g). The clinical presentation can be insidious or fulminant and ranges from apnea, abdominal distension, and bloody stools to intestinal perforation, peritonitis, sepsis, shock, and death.^1^ Therefore, one of the greatest challenges for neonatologists is to identify early reliable clinical signs and symptoms of NEC.^2^ The term “NEC” usually reflects a series of intestinal lesions, which differ in clinical manifestations, pathogenesis, and prevention strategies.^3^, ^4^ One strategy to prevent or treat NEC would be to develop an early diagnostic tool allowing the identification of preterm infants either at the risk of developing NEC or at the onset of symptoms to aid in the diagnostic dilemma and treatment. NEC pathophysiology is generally hypothesized to be multifactorial; common risk factors include low gestational age at birth, low birth weight, chorioamnionitis, mechanical ventilation, and many more. Research into the pathophysiology of NEC has further uncovered risk factors such as intestinal microbial colonization, microcirculation perfusion, and intestinal immune system maturation.^5^ These factors cannot explain the specific pathogenesis of NEC alone, but taken together, the pathogenesis of NEC may be the result of systemic multiple phylogenetic defects.

RNA‐Seq is a method of transcriptome profiling using deep‐sequencing technologies which also provides a far more precise measurement of levels of transcripts than other methods.^6^ In this study, the RNA‐Seq data from human NEC, NEC self‐control (NEC‐SC), and normal control intestinal tissues were analyzed by bioinformatics tools. The immune infiltration in NEC was analyzed by performing the CIBERSORT algorithm method, which is widely used to assess the relative content of 22 kinds of immune cells.^7^

## 2 MATERIALS AND METHODS

### 2.1 Human small intestine

Ethical approval for this study was obtained from the Institutional Review Board of the Children's Hospital of Chongqing Medical University (File No. 2022#345). All methods used in the study were carried out in accordance with approved guidelines and regulations. None of the researchers who analyzed these samples had access to personally identifiable patient information except for the principal investigator (Zhuojun Xie) and the study coordinator (Quan Kang).

Infants with NEC Bell‐IIB stage and above and infants with congenital intestinal stenosis or atresia who were hospitalized and underwent surgery in the Department of General Trauma Surgery, Children's Hospital of Chongqing Medical University from December 2021 to December 2022 were enrolled. At last, 12 preterm infants' intestinal tissues for transcriptome sequencing were collected and three groups were set up: NEC, NEC‐SC, and normal control (NOR). Among them, NEC (*n* = 4) and NEC‐SC (*n* = 3) were from children who were clinically diagnosed with NEC (≥II stage) and underwent intestinal resection in the acute active stage, two tissue sections (NEC lesion and adjacent normal regions) from the resected small bowel segment were collected as follows: (1) an NEC lesion that showed perforation or necrosis and (2) adjacent normal tissue. NOR (*n* = 5) was derived from the normal part of the ileum, which was clinically diagnosed as non‐NEC (such as congenital enterostenosis and intestinal atresia, etc.). Written consent was obtained from the parents/legal guardians of the children for all samples and the objects participated only on the day of surgery, then we will do a postoperative follow‐up 1 year later.

### 2.2 Total RNA extraction and RNA‐Seq

Total RNA was extracted from the tissues using Trizol (Invitrogen, Carlsbad, CA, USA) according to the manual instruction. We used the R package “RNASeqPower” to determine whether we obtained sufficient power to detect patterns of differential gene expression between comparisons of RNA‐Seq data and the statistical power were listed in Table S1. These results demonstrated that the experiments were reproducible and that the data were accurate. RNA‐Seq was assisted by BGI.The sequencing data were filtered with SOAPnuke (v1.5.2, https://github.com/BGI‐flexlab/SOAPnuke) by (1) removing reads containing sequencing adapter, (2) removing reads whose low‐quality base ratio (base quality less than or equal to 5) is more than 20%, and (3) removing reads whose unknown base ('N' base) ratio is more than 5%, afterward clean reads were obtained and stored in FASTQ^8^ format. Data availability: RNA‐Seq data are available under project PRJNA925809 (https://www.ncbi.nlm.nih.gov/sra/PRJNA925809).

### 2.3 Gene expression analysis

We used Bowtie2^9^ (v2.2.5, http://bowtie‐bio.sourceforge.net/Bowtie2/index.shtml) to align clean reads to reference sequences to calculate gene alignment rates and then used salmon^10^ (v1.4.0, https://github.com/COMBINE‐lab/salmon) to calculate gene and transcript expression levels. Salmon incorporates a novel two‐phase parallel inference algorithm, a feature‐rich bias model, and an ultra‐fast read mapping program to obtain TPM and NumReads values, which greatly improved the accuracy of abundance estimation and the sensitivity of differential expression analysis. Salmon is a software package for calculating the expression of genes as well as transcripts from RNA‐seq reads. In this project, the cor function in R software was used to calculate the Pearson correlation coefficient between every two samples, the hclust function in R software was used for hierarchical clustering analysis, the princomp function in R software was used for principal component analysis (PCA), and the ggplot2 package in R software was used for drawing graphs.

### 2.4 Identification of DEGs

On demand, we used the DEseq2 algorithm for differential gene detection. The DEseq2 method is based on the principle of negative binomial distribution, and this project performs differentially expressed gene (DEG) detection according to the method described in Love et al.^11^ We used a fold change of two or more and an adjusted *p* value of 0.05 or less to screen DEGs.

### 2.5 GO and KEGG pathway analysis of DEGs

According to the gene ontology (GO) and KEGG annotation results and the official classification, we classified the differential genes into functional classification and biological pathway classification while using the phyper function in R software for enrichment analysis. The *p* value is calculated as follows:

$$p=1-\sum_{i=0}^{m-1}\frac{\left(\begin{array}{c} M\\ i \end{array}\right)\left(\begin{array}{c} N-M\\ n-i \end{array}\right)}{\left(\begin{array}{c} N\\ n \end{array}\right)}$$

The *p* value was then FDR‐corrected, and generally, functions with FDR ≤0.01 were considered significantly enriched.

### 2.6 Validation of RNA‐seq experiments

The RNA samples used for RNA‐seq analyses were subjected to reverse transcription quantitative real‐time PCR (RT‐qPCR) analysis. Each experiment was conducted with three technical replicates. For each sample, reverse transcription of 1 μg total RNA in a 20 μL volume was performed with ABScript III RT Master Mix for qPCR with gDNA remover (ABclonal). The reaction was carried out at 37°C for 2 min, 55°C for 15 min, 85°C for 5 min, and was finally maintained at 4°C. Gene‐specific primers were designed using Sangon Biotech (https://www.sangon.com/primerDesign). The primers used in this study are listed in Table S1. GADPH was selected as an endogenous control. Each qPCR reaction contained 10 μL of 2 × Universal SYBR Green Fast qPCR Mix (ABclonal), 0.8 μL of cDNA sample, and 0.4 μL of forward and reverse primers in a final volume of 20 μL. The cycling conditions were as follows: 95°C for 3 min, 95°C for 5 s, and 60°C for 34 s, 40 cycles. CFX96 real‐time system (BIO‐RAD) was used to analyze the melting point curve to verify the specificity. The relative expression levels were calculated using the 2–ΔΔCt method.^12^

### 2.7 Immune infiltration analysis

The Cibersort package was used for immune cell evaluation based on gene sequencing data. The cytogenetic signature gene set was LM22, which contains a total of 547 genes for 22 immune cells.^7^ ANOVA was used to compare differences between groups. *p* < 0.05 was considered significant.

## 3 RESULTS

### 3.1 Gene expression analysis

**FIGURE 1:**
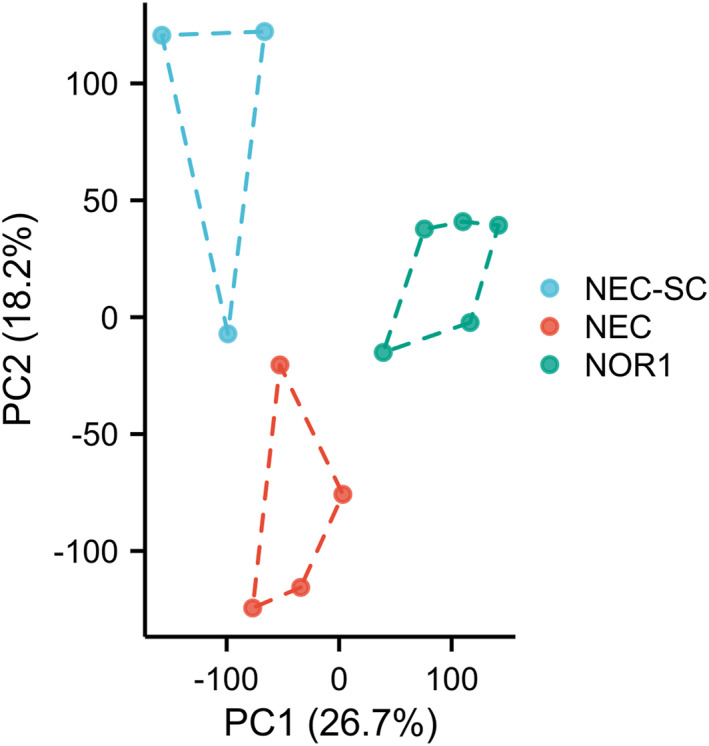
PCA analysis. The *X* and *Y* axes represent the new dataset corresponding to the principal components obtained after the dimension reduction of the sample expression, which is used to represent the gap between samples. The values in the parentheses of the axis labels represent the percentage of the total variance explained by the corresponding principal components. PCA, principal component analysis.

In the analysis of transcriptome, PCA (Figure 1) reduces the dimension of a large number of gene expression information contained in a sample to a few unrelated principal components, so as to make comparison between samples, find out outlier samples, and identify sample clusters with high similarity.

### 3.2 Identification of DEGs

**FIGURE 2:**
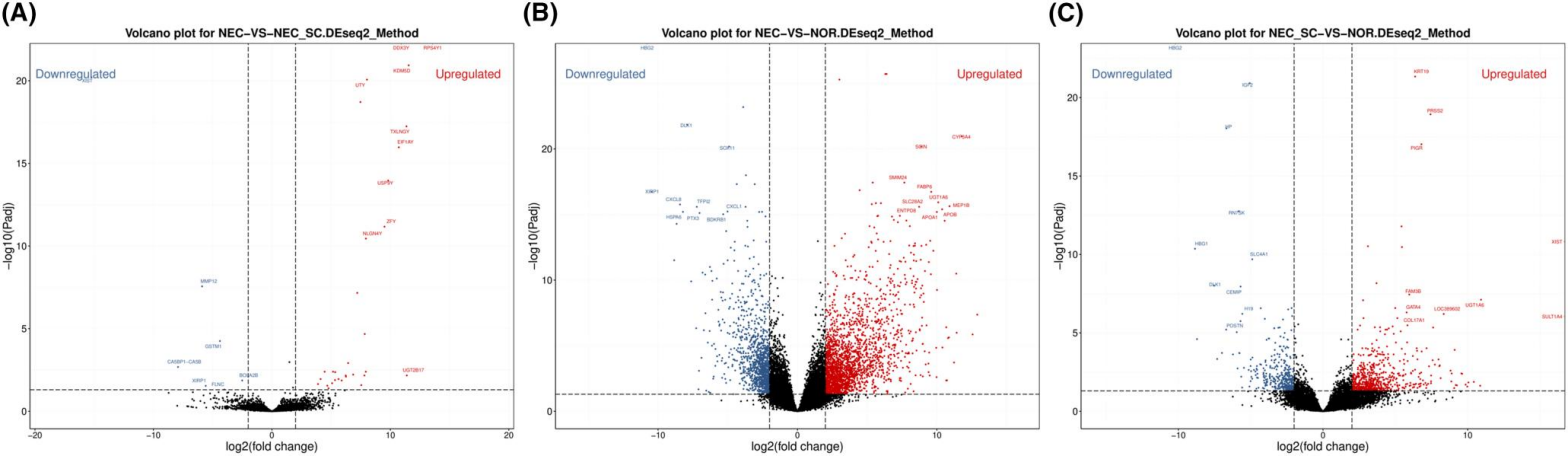
Volcano‐plot distribution map of DEG. The *X* axis represents the log2 transformed difference fold value, and the *Y* axis represents the −log10 transformed significance value. Red represents upregulated DEGs, blue represents downregulated DEGs, and gray represents non‐DEGs. DEGs, differentially expressed genes. (A–C) Vocano plots for NEC vs NEC‐SC, NEC vs NOR, NEC‐SC vs NOR.

According to the gene expression level of each sample, we can detect DEG between samples (groups). A total of 34,712 genes were detected in this study, of which 33 genes were upregulated and 7 genes were downregulated in NEC versus NEC‐SC group, 3465 genes were upregulated and 2499 genes were downregulated in NEC versus NOR group, and 846 genes were upregulated and 613 genes were downregulated in NEC‐SC versus NOR group. Volcano plots (Figure 2A–C, Tables 1, 2, 3) were used to show the distribution of DEGs, and the top 10 DEGs with the most significant differences were marked. Among these DEGs, by comparing the DEG datasets, we found that MMP12, BOLA2B, KDM5D, and RPS4Y1 were significantly different in NEC versus NEC‐SC (*p* < 0.001) but not in NEC‐SC versus NOR (*p* > 0.05), which suggests that these DEGs in self‐control tissues are not affected by necrotizing invasion of NEC, and they may become new potential genes to promote the study of the pathogenesis of NEC.

**TABLE 1:** NEC‐VS‐NEC_SC.DEseq2_Method DEGs (up‐/down‐top 10).

Up‐/down‐regulation	Gene	Exp (NEC)	Exp (NEC‐SC)	*p* value
Up	*DDX3Y*	1.32	2539.46	2.32E‐42
Up	*RPS4Y1*	0.23	3896.23	6.15E‐34
Up	*KDM5D*	0.57	1577.23	1.99E‐25
Up	*UTY*	1.87	480.81	2.45E‐24
Up	*TXLNGY*	0	510.84	2.30E‐21
Up	*EIF1AY*	0.28	537.37	4.83E‐20
Up	*USP9Y*	0.54	474.86	5.64E‐18
Up	*ZFY*	0.23	232.86	3.84E‐15
Up	*NLGN4Y*	1.07	258.45	2.23E‐14
Up	*UGT2B17*	0.28	858.04	9.70E‐06
Down	*XIST*	6350.22	0	1.42E‐24
Down	*MMP12*	1934.34	32.56	1.89E‐11
Down	*GSTM1*	345.54	16.48	4.81E‐08
Down	*CA5BP1‐CA5B*	40.15	0	2.15E‐06
Down	*BOLA2B*	63.67	11.17	2.48E‐05
Down	*XIRP1*	6042.08	125.65	6.12E‐05
Down	*FLNC*	78,232.18	4805.83	0.00011

*Note*: Exp: gene expression level.

**TABLE 2:** NEC‐VS‐NOR.DEseq2_Method DEGs (up‐/down‐top 10).

Up‐/down‐regulation	Gene	Exp (NEC)	Exp (NOR)	*p* value
Up	*CYP3A4*	5.77	20,553.47	4.69E‐25
Up	*SCIN*	8.35	3898.79	3.51E‐24
Up	*SMIM24*	49.79	10,135.08	2.38E‐21
Up	*FABP6*	8.96	6934.31	1.77E‐20
Up	*UGT1A6*	0.8	834.72	1.19E‐19
Up	*MEP1B*	4.02	510.84	2.96E‐19
Up	*SLC28A2*	5.91	2505.45	3.66E‐19
Up	*APOB*	54.26	72,268.72	5.93E‐19
Up	*APOA1*	153.45	157,087.7	1.09E‐18
Up	*ENTPD8*	12.91	2109.63	2.46E‐18
Down	*HBG2*	23,566.92	13.37	1.45E‐37
Down	*DLK1*	337.57	1.36	5.25E‐26
Down	*SOX11*	469.1	15.42	3.30E‐24
Down	*XIRP1*	5751.09	4.11	1.68E‐20
Down	*CXCL8*	19,302	56.07	2.09E‐19
Down	*TFPI2*	2709.99	18.1	3.71E‐19
Down	*CXCL1*	2175.36	67.12	9.10E‐19
Down	*HSPA6*	16,721.91	56.24	1.03E‐18
Down	*PTX3*	3886.64	29.8	1.39E‐18
Down	*BDKRB1*	1862.96	46.34	1.77E‐18

*Note*: Exp: gene expression level.

**TABLE 3:** NEC_SC‐VS‐NOR.DEseq2_Method DEGs (up‐/down‐top 10).

Up‐/down‐regulation	Gene	Exp (NEC‐SC)	Exp (NOR)	*p* value
Up	*KRT19*	208.85	17,262.75	4.88E‐26
Up	*PRSS2*	19.23	3323.65	2.50E‐23
Up	*PIGR*	196.17	21,919.36	3.04E‐21
Up	*XIST*	0	2490.92	7.46E‐15
Up	*FAM3B*	40.13	2503.89	3.28E‐11
Up	*UGT1A6*	0.45	869.59	7.46E‐11
Up	*GATA4*	11.94	655.45	6.44E‐10
Up	*LOC389602*	1.33	434.92	8.76E‐10
Up	*SULT1A4*	0	715.31	1.37E‐09
Up	*COL17A1*	69.98	4002.9	6.22E‐09
Down	*HBG2*	16,595.75	14.01	8.40E‐48
Down	*IGF2*	20,916.7	622.91	1.93E‐25
Down	*HP*	424.27	4.05	2.45E‐22
Down	*RN7SK*	402.75	7.13	6.56E‐17
Down	*HBG1*	4960.77	10.68	2.81E‐14
Down	*SLC4A1*	99.36	3.35	1.43E‐13
Down	*DLK1*	267.93	1.43	7.81E‐12
Down	*CEMIP*	1482.97	28.47	9.65E‐12
Down	*H19*	38,891.53	813.42	8.28E‐10
Down	*POSTN*	15,425.83	295.91	3.18E‐09

*Note*: Exp: gene expression level.

### 3.3 GO function enrichment analysis of the DEGs

According to the results of GO analysis, the GO function of the DEGs was classified and enriched. GO is divided into three functional categories: molecular function, cellular component, and biological process. We listed the top five GO terms of each category with the highest enrichment degree. The results showed that DEGs in NEC versus NEC‐SC group were mainly involved in CCR1 chemokine receptor binding (GO:0031726), brush border (GO:0005903), cholesterol metabolic process (GO:0008203), etc. (Table 4). DEGs in NEC versus NOR group were mainly involved in transporter activity (GO:0005215), cell periphery (GO:0071944), regulation of biological quality (GO:0065008), etc. (Table 5). DEGs in the NEC‐SC versus NOR group were mainly involved in growth factor binding (GO:0019838), extracellular region (GO:0005576), extracellular matrix organization (GO:0030198), etc. (Table 6).

**TABLE 4:** GO analyses results of DEGs (top 5 of each ontology according to corrected *p* value). NEC‐VS‐NEC_SC.

Ontology	ID	GO term	Corrected *p* value	Gene ID
Molecular	GO:0031726	CCR1 chemokine receptor binding	0.00976	9560, 6352
Molecular	GO:0031730	CCR5 chemokine receptor binding	0.00976	9560, 6352
Cellular	GO:0005903	Brush border	8.15e‐05	4311, 1811, 340024, 11136, 8029
Cellular	GO:0031526	Brush border membrane	0.00037	1811, 340024, 11136, 8029
Cellular	GO:0098862	Cluster of actin‐based cell projections	0.00059	4311, 1811, 340024, 11136, 8029
Cellular	GO:0034363	Intermediate‐density lipoprotein particle	0.00146	335, 338
Cellular	GO:0034362	Low‐density lipoprotein particle	0.01885	335, 338
Biological	GO:0008203	Cholesterol metabolic process	0.02543	335, 8029, 338, 6822
Biological	GO:1902652	Secondary alcohol metabolic process	0.03464	335, 8029, 338, 6822
Biological	GO:0008202	Steroid metabolic process	0.04226	335, 7367, 8029, 338, 6822
Biological	GO:0016125	Sterol metabolic process	0.04359	335, 8029, 338, 6822

Abbreviations: DEGs, differentially expressed genes; GO, gene ontology.

**TABLE 5:** GO analyses results of DEGs (top 5 of each ontology according to corrected *p* value). NEC‐VS‐NOR.

Ontology	ID	GO term	Corrected *p* value	Cluster frequency
Molecular	GO:0005215	Transporter activity	3.39e‐09	395 out of 3980 genes, 9.9%
Molecular	GO:0022857	Transmembrane transporter activity	1.12e‐08	363 out of 3980 genes, 9.1%
Molecular	GO:0005515	Protein binding	7.08e‐07	1923 out of 3980 genes, 48.3%
Molecular	GO:0015075	Ion transmembrane transporter activity	1.89e‐06	315 out of 3980 genes, 7.9%
Molecular	GO:0008509	Anion transmembrane transporter activity	6.65e‐06	169 out of 3980 genes, 4.2%
Cellular	GO:0071944	Cell periphery	3.84e‐38	1794 out of 4416 genes, 40.6%
Cellular	GO:0005886	Plasma membrane	8.53e‐31	1630 out of 4416 genes, 36.9%
Cellular	GO:0098590	Plasma membrane region	9.15e‐22	443 out of 4416 genes, 10.0%
Cellular	GO:0031224	Obsolete intrinsic component of membrane	1.43e‐21	1660 out of 4416 genes, 37.6%
Cellular	GO:0031226	Obsolete intrinsic component of plasma membrane	1.01e‐20	589 out of 4416 genes, 13.3%
Biological	GO:0065008	Regulation of biological quality	8.14e‐24	1192 out of 4114 genes, 29.0%
Biological	GO:0032879	Regulation of localization	1.21e‐20	855 out of 4114 genes, 20.8%
Biological	GO:0042221	Response to chemical	1.47e‐19	1151 out of 4114 genes, 28.0%
Biological	GO:0050896	Response to stimulus	6.78e‐19	2147 out of 4114 genes, 52.2%
Biological	GO:0006629	Lipid metabolic process	7.58e‐19	441 out of 4114 genes, 10.7%

Abbreviations: DEGs, differentially expressed genes; GO, gene ontology.

**TABLE 6:** GO analyses results of DEGs (top 5 of each ontology according to corrected *p* value). NEC_SC ‐VS‐NOR.

Ontology	ID	GO term	Corrected *p* value	Cluster frequency
Molecular	GO:0019838	Growth factor binding	1.28e‐09	34 out of 1022 genes, 3.3%
Molecular	GO:0005201	Extracellular matrix structural constituent	3.37e‐09	33 out of 1022 genes, 3.2%
Molecular	GO:0030020	Extracellular matrix structural constituent conferring tensile strength	2.17e‐06	13 out of 1022 genes, 1.3%
Molecular	GO:0048407	Platelet‐derived growth factor binding	9.80e‐06	8 out of 1022 genes, 0.8%
Molecular	GO:0005178	Integrin binding	0.00022	27 out of 1022 genes, 2.6%
Cellular	GO:0005576	Extracellular region	7.32e‐26	400 out of 1157 genes, 34.6%
Cellular	GO:0005615	Extracellular space	1.86e‐24	325 out of 1157 genes, 28.1%
Cellular	GO:0031012	Extracellular matrix	1.39e‐21	104 out of 1157 genes, 9.0%
Cellular	GO:0030312	External encapsulating structure	1.62e‐21	104 out of 1157 genes, 9.0%
Cellular	GO:0071944	Cell periphery	1.84e‐19	526 out of 1157 genes, 45.5%
Biological	GO:0030198	Extracellular matrix organization	1.19e‐20	83 out of 1072 genes, 7.7%
Biological	GO:0043062	Extracellular structure organization	1.75e‐20	83 out of 1072 genes, 7.7%
Biological	GO:0045229	External encapsulating structure organization	2.11e‐20	83 out of 1072 genes, 7.7%
Biological	GO:0001568	Blood vessel development	2.93e‐17	90 out of 1072 genes, 8.4%
Biological	GO:0001944	Vasculature development	5.47e‐17	92 out of 1072 genes, 8.6%

Abbreviations: DEGs, differentially expressed genes; GO, gene ontology.

### 3.4 KEGG pathway enrichment analysis of the DEGs

**FIGURE 3:**
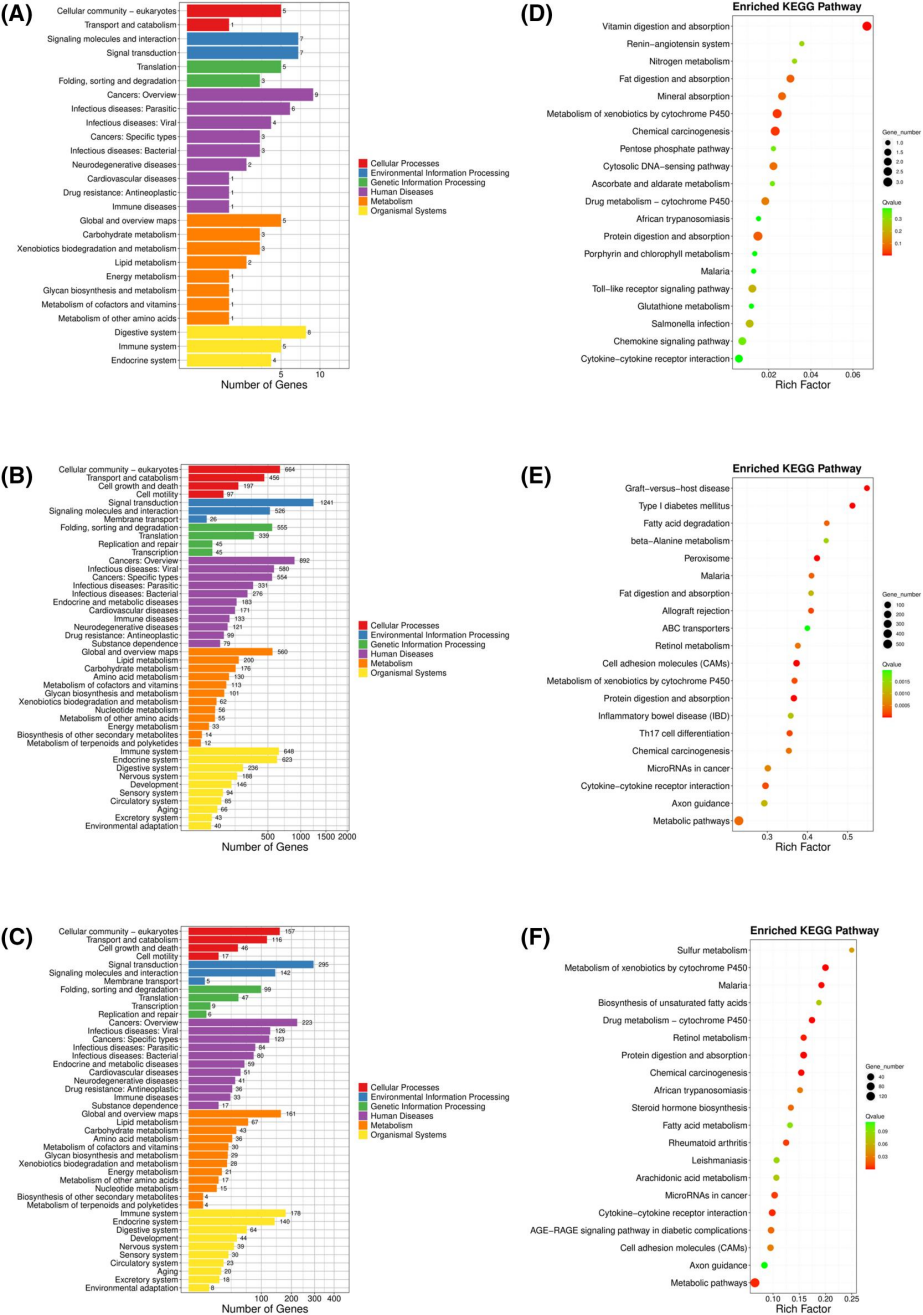
KEGG pathway classification and enrichment analysis between NEC, NEC‐SC and NOR. (A–C) The *X* axis represents the proportion of genes and the *Y* axis represents the KEGG functional classification. The genes were divided into 7 branches according to the KEGG metabolic pathways involved: cellular processes, environmental information processing, genetic information processing, human disease (animals only), metabolism, organismal systems, and drug development. (D–F) The *X* axis represents enrichment factor values and the *Y* axis represents pathway names. The color represents the *Q* value (the whiter the color, the larger the value; the bluer the color, the smaller the value), and the smaller the value, the more significant the enrichment result. The size of the dots represents the number of DEGs (larger dots represent larger numbers, and smaller dots represent smaller numbers). Rich Factor refers to the enrichment factor value, which is the quotient of the foreground value (number of differentially expressed genes) of a pathway on annotation and the background value (number of all genes) of a pathway on annotation. The larger the data, the more obvious the enrichment result. (A, D: NEC vs. NEC‐SC; B,E: NEC vs. NOR; C, F: NEC‐SC vs. NOR). DEGs, differentially expressed genes; NEC, necrotizing enterocolitis.

KEGG biological pathway classification results are shown in Figure 3A–C and partial pathway enrichment results are shown in Figure 3D–F. The results show that in the NEC versus NEC‐SC group, DEGs were mainly enriched in the toll‐like receptor signaling pathway, etc. In the NEC versus NOR group, DEGs were mainly enriched in Th17 cell differentiation, etc. In NEC‐SC versus NOR group, DEGs were mainly enriched in cytokine–cytokine receptor interaction, etc. Meanwhile, the top 10 pathways with the highest enrichment and their corresponding number of DEGs are shown in Tables 7, 8, 9. It is worth noting that in the three comparison groups, DEGs were mainly enriched in the metabolism of xenobiotics by cytochrome P450.

**TABLE 7:** Pathway analyses results of DEGs (top 10 according to *Q* value). NEC‐VS‐NEC_SC.

#Pathway	DEGs genes with pathway annotation (37)	All genes with pathway annotation (25,374)	*p* value	*Q* value	Pathway ID
Vitamin digestion and absorption	3 (8.11%)	45 (0.18%)	3.88249e‐05	0.003028342	ko04977
Metabolism of xenobiotics by cytochrome P450	3 (8.11%)	125 (0.49%)	0.0008023798	0.023371465	ko00980
Chemical carcinogenesis	3 (8.11%)	130 (0.51%)	0.0008989025	0.023371465	ko05204
Protein digestion and absorption	3 (8.11%)	202 (0.8%)	0.003164424	0.061706268	ko04974
Fat digestion and absorption	2 (5.41%)	66 (0.26%)	0.004184726	0.065281726	ko04975
Mineral absorption	2 (5.41%)	76 (0.3%)	0.005509504	0.071623552	ko04978
Cytosolic DNA‐sensing pathway	2 (5.41%)	90 (0.35%)	0.007643903	0.085174919	ko04623
Drug metabolism—cytochrome P450	2 (5.41%)	109 (0.43%)	0.01104092	0.107648970	ko00982
Toll‐like receptor signaling pathway	2 (5.41%)	164 (0.65%)	0.0238489	0.206690467	ko04620
*Salmonella* infection	2 (5.41%)	183 (0.72%)	0.02920668	0.227812104	ko05132

*Note*: *Q* value: The corrected *p* value, a smaller *Q* value indicates a more significant difference.

Abbreviation: DEGs, differentially expressed genes.

**TABLE 8:** Pathway analyses results of DEGs (top 10 according to *Q* value). NEC‐VS‐NOR.

#Pathway	DEGs genes with pathway annotation (4981)	All genes with pathway annotation (25,374)	*p* value	*Q* value	Pathway ID
CAMs	94 (1.89%)	252 (0.99%)	4.579001e‐11	1.479017e‐08	ko04514
Type I diabetes mellitus	42 (0.84%)	82 (0.32%)	1.580471e‐10	2.552461e‐08	ko04940
Graft‐versus‐host disease	34 (0.68%)	62 (0.24%)	8.191342e‐10	8.819345e‐08	ko05332
Peroxisome	53 (1.06%)	125 (0.49%)	4.710647e‐09	3.803847e‐07	ko04146
Protein digestion and absorption	74 (1.49%)	202 (0.8%)	1.240844e‐08	8.015852e‐07	ko04974
Allograft rejection	36 (0.72%)	88 (0.35%)	3.63317e‐06	1.744727e‐04	ko05330
Th17 cell differentiation	53 (1.06%)	149 (0.59%)	3.781143e‐06	1.744727e‐04	ko04659
Cytokine–cytokine receptor interaction	102 (2.05%)	345 (1.36%)	5.674203e‐06	2.054833e‐04	ko04060
Metabolism of xenobiotics by cytochrome P450	46 (0.92%)	125 (0.49%)	5.72554e‐06	2.054833e‐04	ko00980
Fatty acid degradation	26 (0.52%)	58 (0.23%)	1.141013e‐05	3.385554e‐04	ko00071

*Note*: *Q* value: The corrected *p* value, a smaller *Q* value indicates a more significant difference.

Abbreviations: CAMs, cell adhesion molecules; DEGs, differentially expressed genes.

**TABLE 9:** Pathway analyses results of DEGs (top 10 according to *Q* value). NEC_SC‐VS‐NOR.

#Pathway	DEGs genes with pathway annotation (1238)	All genes with pathway annotation (25,374)	*p* value	*Q* value	Pathway ID
Metabolism of xenobiotics by cytochrome P450	25 (2.02%)	125 (0.49%)	1.514801e‐09	4.665587e‐07	ko00980
Protein digestion and absorption	32 (2.58%)	202 (0.8%)	3.984597e‐09	6.136279e‐07	ko04974
Drug metabolism—cytochrome P450	19 (1.53%)	109 (0.43%)	1.271948e‐06	1.305867e‐04	ko00982
Malaria	15 (1.21%)	78 (0.31%)	4.688104e‐06	3.091233e‐04	ko05144
Chemical carcinogenesis	20 (1.62%)	130 (0.51%)	5.018236e‐06	3.091233e‐04	ko05204
Retinol metabolism	16 (1.29%)	101 (0.4%)	2.984243e‐05	1.531911e‐03	ko00830
Metabolic pathways	157 (12.68%)	2394 (9.43%)	7.173965e‐05	3.156545e‐03	ko01100
Cytokine‐cytokine receptor interaction	34 (2.75%)	345 (1.36%)	8.590352e‐05	3.307286e‐03	ko04060
MicroRNAs in cancer	27 (2.18%)	262 (1.03%)	0.0002156411	7.203079e‐03	ko05206
Rheumatoid arthritis	18 (1.45%)	144 (0.57%)	0.0002338662	7.203079e‐03	ko05323

*Note*: *Q* value: The corrected *p* value, a smaller *Q* value indicates a more significant difference.

Abbreviation: DEGs, differentially expressed genes.

### 3.5 RT–PCR validation of the DEGs

**FIGURE 4:**
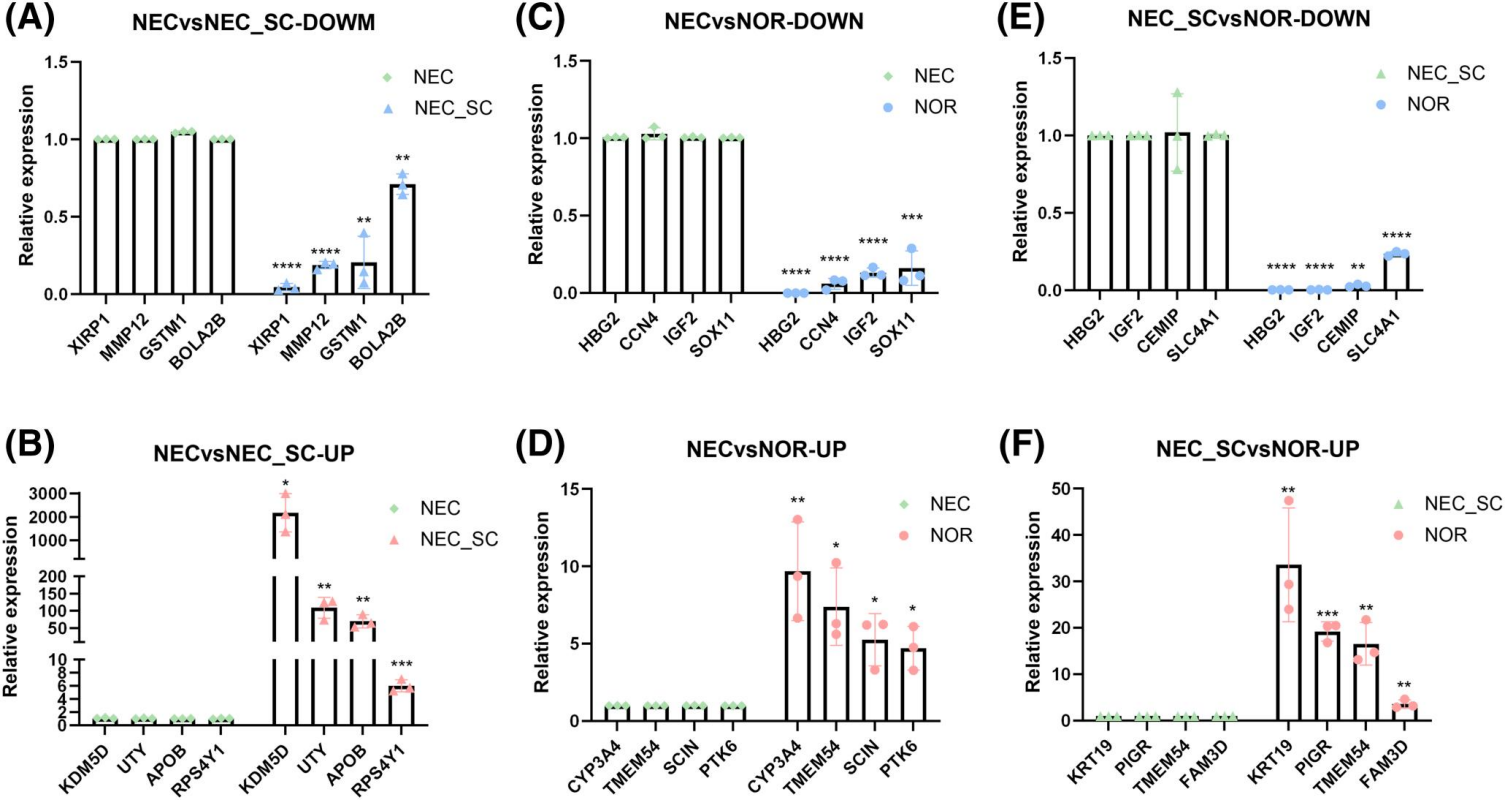
qPCR validation of DEGs. DEGs, differentially expressed genes; qPCR, quantitative real‐time PCR. *p < 0.05, **p < 0.01, ***<0.001, ****p < 0.0001; *n* = 3. (A–B) down/up regulation of NEC vs NEC‐SC; (C–D) down/up regulation of NEC vs NOR; (E–F) down/up regulation of NEC‐SC vs NOR.

Based on the identified DEGs, 21 DEGs were selected from the significance of the fold change greater than or equal to 5 times, and these DEGs have been reported to be differentially expressed in intestinal inflammation, regeneration, or other diseases in the current literature. In addition, the results showed that the relative expression levels of the 21 DEGs (HBG2, CCN4, IGF2, SOX11, CYP3A4, TEME54, SCIN, PTK6, XIRP1, MMP12, GSTM1, BOLA2B, KDM5D, UTY, AOPB, RPS4Y1, CEMIP, SLC4A1, KRT19, PIGR, and FAM3D) were consistent with the sequencing results (Figure 4A–F, *p* < 0.05), which indicated that the sequencing results were accurate and could be used for subsequent functional analysis.

### 3.6 Immune infiltration analyses

**FIGURE 5:**
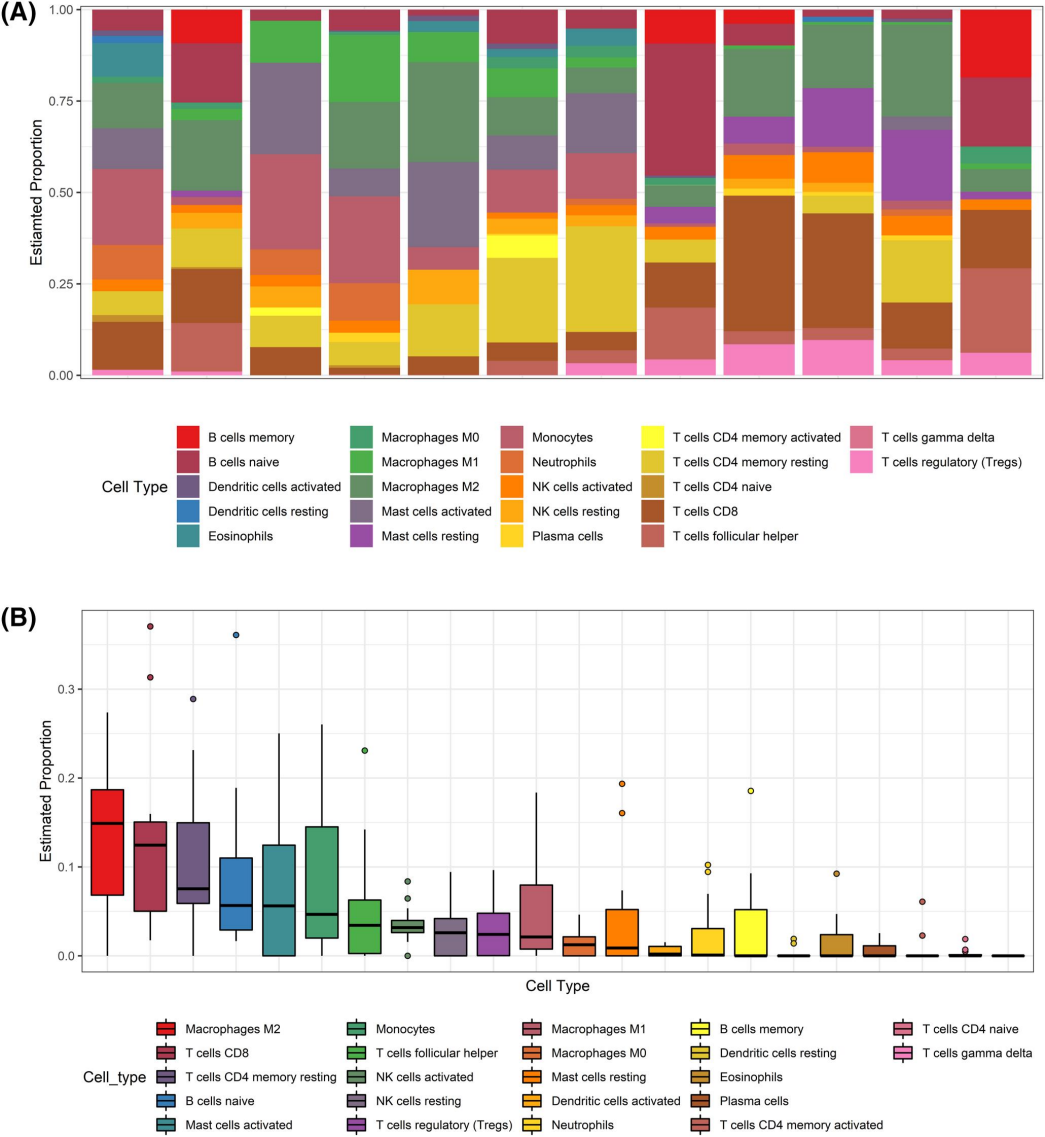
Heatmap and box plot of immune cell infiltration. (A) proportion of immune cells in each sample. (B) proportion of immune cells in all samples.

**FIGURE 6:**
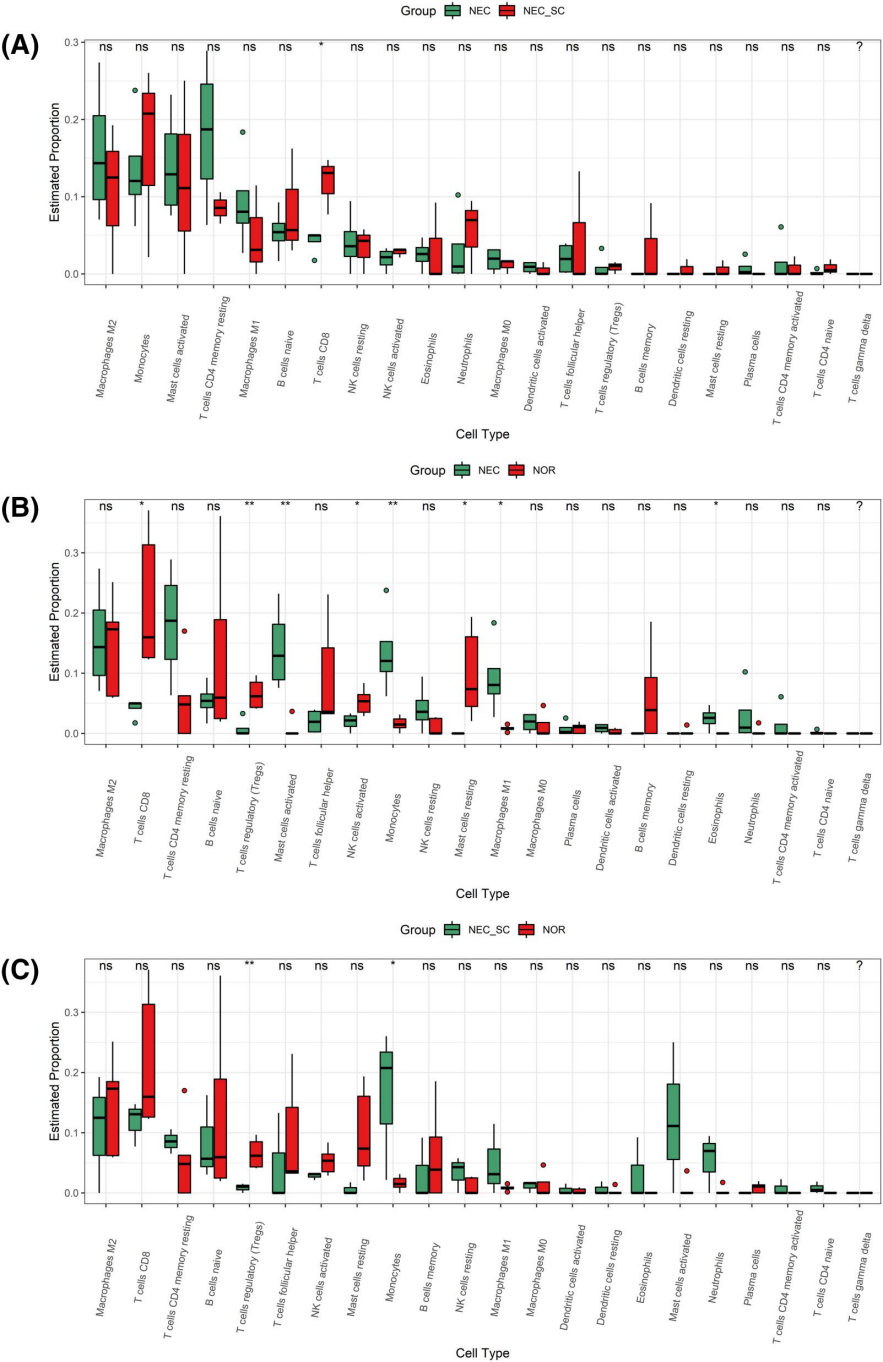
Differences in immune cell infiltration among groups. *p < 0.05, **p < 0.01, ns: no significance; *n* = 3. (A–C) immune infiltration of NEC vs NEC‐SC, NEC vs NOR and NEC‐SC vs NOR.

The immune infiltration of NEC has not been fully revealed, especially in neonates whose immune systems have not yet developed. Firstly, we investigated the differences in immune infiltration between NEC, NEC‐SC, and normal intestinal tissues in 22 immune cell subsets. Results from five normal controls and four NEC patients as well as three self‐controls are summarized in Figure 5A,B. NEC intestinal tissue generally contained a lower proportion of CD8+T cells than its own controls (Figure 6A, *p* < 0.05). Compared with normal control tissue, NEC intestinal tissue contained a higher proportion of activated mast cells monocytes, M1 Macrophages, and eosinophils, and there were a low proportion of CD8+T cells, T cells regulatory (Tregs), NK cells activated, and Mast cells resting (Figure 6B, *p* < 0.05). Compared with normal tissues, the self‐controlled intestinal tissues of NEC contained a lower proportion of T cells regulatory (Tregs) and a higher proportion of monocytes (Figure 6C, *p* < 0.05).

## 4 DISCUSSION

To understand how gut homeostasis is maintained and regulated based on the complex interplay between intestinal epithelial cells, the immune system, and the microbiota are areas of intense research. Recently, some other RNA‐Seq for gene expression profiling in human NEC were reported.^13^, ^14^ Their results identified several potential genes that contribute to the development of NEC (such as DPF3, CAMK4, and PCP4), when compared to our results, PCP4 was overlapped. However, their research omitted the comparison between the NEC's own controls (the normal tissue adjacent to the necrotic intestinal segment) and the normal controls, and we could not determine whether the detection of differences between them would be affected if the adjacent tissues of NEC were invaded by lesions. In our study, we performed transcriptome sequencing of all three intestinal tissues and pairwise comparison and analysis, which will help us to understand more deeply the pathogenesis of NEC at the molecular pathological level. The results of DEGs in the NEC group, NEC‐SC group, and NOR group showed that in addition to a series of recognized risk factors such as preterm, birth weight, feeding intolerance, and so on, not only the necrotic intestinal segment but also the non‐pathological intestinal segment of NEC patients had differential changes at the gene level compared with the normal intestinal segment during the development of NEC. This difference may be established before birth and seems to explain the current disagreement between clinicians and experts about the importance of different risk factors.^15^ In this study, we hypothesized that global gene expression profiling may reveal distinct genetic differences between NEC lesion, adjacent normal region, and the normal control. Although our study did not go deeper or more specific, several possible potential genes (MMP12, BOLA2B, KDM5D, and RPS4Y1) were identified (*p* < 0.001). As described in the results, it is suggested that these DEGs may be the potential genes affecting the development of NEC and their differential expression in NEC‐SC may not be due to the invasion of necrotic tissues as they are not different from normal controls, and on the contrary, it may rule out the influence of other enteropathies (such as enterostenosis) in the normal controls. However, further replication and evaluation studies are needed.

Matrix metalloproteinases (MMP) are involved in tissue remodeling and cell migration, both being important aspects of inflammatory disease. Metalloelastase MMP‐12 is upregulated in NEC,^16^ which is consistent with the results of our study. Downregulation of KDM5D in CRC patients was associated with poor prognoses. Overexpression of KDM5D significantly inhibited the growth and metastasis of CRC in vitro and in vivo.^17^ It is worth noting that in our study, the NEC‐SC group exhibited a high expression of KDM5D gene, which may be one of the mechanisms to rescue intestinal necrosis. In the case of BOLA2B and RPS4Y1, although a direct association between NEC and BOLA2B or RPS4Y1 has not been reported, functional analysis showed that BOLA2B was enriched in pathways associated with cell cycle inhibition,^18^ which may inhibit intestinal cell proliferation leading to disruption of the intestinal barrier and thereby increase the severity of NEC. Here, a brief description is given for the other DEGs. The intestinal epithelial cells are in constant turnover and are replenished by intestinal stem cells (ISC) that express the Leucine‐rich repeat‐containing G‐protein coupled receptor 5 (Lgr5). Krt19 transcript localizes to the stem‐cell zone above the crypt base and marks both colonic and ISC, and the distinct nature of Krt19+ versus Lgr5+ stem cells was confirmed by the observation that Krt19+ cells continue to lineage trace crypts despite the ablation of Lgr5+ stem cells in both the colon and intestine. ISC depletion correlates with severe gut damage during NEC development.^19^, ^20^ PIGR, which encodes a key molecule for the transcytosis of immunoglobulin A is one such gene and is known to be downregulated in the colorectal epithelium in IL‐17R‐deficient mice and in patients with inflammatory bowel disease (IBD)^21^ and is also found to be downregulated in NEC‐SC in our study. After the verification of the top 4 DEGs in each comparison group, their relative expression levels were consistent with the sequencing results, indicating that our sequencing data results were reliable in this study. Some of these DEGs may be closely related to the occurrence and development of NEC. Some studies have found that CYP3A4 is the most abundant CYP enzyme in the small intestine, which is crucial for intestinal biotransformation^22^ and its loss (low expression) may be one of the reasons for the development and progression of NEC. The actin‐severing protein scinderin (SCIN) is required for cell proliferation and its downregulation inhibits cell proliferation as well as WNT/β‐catenin signaling pathway. Notably, reduced WNT pathway activity has been found to inhibit the proliferation of ISC, leading to the development of NEC.^20^, ^23^ FAM3D represents a class of guardians of the gut; the deficiency in FAM3D is associated with impaired integrity of colonic mucosa, increased epithelial hyper‐proliferation, reduced antimicrobial peptide production, and increased sensitivity to chemically induced colitis.^24^

Based on the KEGG database, the DEGs were mainly enriched in the toll‐like receptor signaling pathway, IBD, rheumatoid arthritis pathway, and other inflammatory response pathways. The results indicated that NEC was characterized by inflammatory processes in the process of disease. It is worthy to note that in the three comparison groups, we found that DEGs were significantly enriched in the metabolism of xenobiotics by cytochrome P450. Cytochromes are expressed in many different tissues of the human body. They are found mostly in intestinal tissues, cytochromes P450 (CYPs) are enzymes that oxidize substances using iron and are able to metabolize a large variety of xenobiotic substances.^25^ CYP enzymes are implicated in tissue growth, development, and ontogeny and are associated with pronounced changes in the microbiota.^26^ It was shown that tremendous metabolic activity of microbiota is associated with higher levels of bacterial CYP enzymes.^27^ Activity and expression of CYP enzymes are significantly affected by microbiota composition, infections, and inflammation, especially by inflammatory cytokines.^28^ However, the direct role of CYP1A1 beyond intestinal disorders such as NEC are yet to be explored. The pathogenesis of NEC is multifactorial resulting from combinations of genetic polymorphism, feeding strategy, altered microbiota, and dysregulation of the immune system.^29^

Major factors in the development of NEC also include functional and anatomical immaturity of the intestinal barrier and intestinal immune system as well as abnormal colonization of intestinal microbes.^30^ The intestine represents the largest compartment of the immune system. Intestinal immune processes are also increasingly implicated in controlling disease development elsewhere in the body.^31^ Therefore, in this study, we also analyzed the differences in immune infiltration between the three groups of intestinal tissues, which may provide a preliminary molecular rationale for the mechanism by which defects in the development of the immune system in preterm infants lead to the development and progression of NEC.

However, our study also has several limitations such as insufficient sample size and lack of functional evaluation. Although the incidence of NEC is high, the limited study time and the limited number of clinically available samples for sequencing may have contributed to the small sample size. Also, this study used ileum tissues from preterm patients with other diseases for the normal control, we do not rule out the possible effect of congenital diseases (such as enterostenosis, intestinal atresia, etc.). Recent discoveries have shed light on a unifying theorem to explain the pathogenesis of NEC, suggesting that specific treatments might finally be forthcoming.

## 5 CONCLUSIONS

In our study, a total of 7463 DEGs were identified. The expression levels of 21 hub genes were confirmed by RT–PCR. Some of these genes (such as MMP12, KDM5D, and Krt19) have been confirmed to be closely related to the occurrence and development of NEC, and our results provide further theoretical basis for the selection of gene therapy targets in the future. The biological functions and pathways of the identified genes provide a more detailed molecular mechanism for understanding NEC development and also provide new ideas and insights for future research. GO function and KEGG pathway analysis provided us with more research targets related to the pathogenesis and regulation mechanism of NEC. And most importantly, by coupling reliable deconvolution algorithms with large‐scale genomic data, we found significant differences in immune infiltration between NEC, NEC‐SC, and normal controls. However, further studies are needed to confirm the relationship between these key genes and immune infiltration as well as their roles in the development of NEC and immune infiltration spectrum.

## AUTHOR CONTRIBUTIONS

Zhuojun Xie and Quan Kang conceived and designed the experiments, and the experiments were performed by Zhuojun Xie and Junbao Du. Data analysis was mainly done by Zhuojun Xie, Yulu Shi, and Hao Jiang. Figures and tables were prepared by Zhuojun Xie, Yulu Shi, Junbao Du, and Hao Jiang. Zhuojun Xie drafted the work, and Quan Kang revised it critically for important content.

## CONFLICT OF INTEREST STATEMENT

All authors have no potential conflicts of interest.

## ETHICS STATEMENT

Ethical approval for this study was obtained from the Institutional Review Board of the Children's Hospital of Chongqing Medical University (File No. 2022#345).

## Supporting information

Table S1

## Data Availability

The data that support the findings of this study are openly available in National Library of Medicine at https://www.ncbi.nlm.nih.gov/.
