# Supplementary material for: A transcriptomic landscape analysis of human necrotizing enterocolitis: Important roles of immune infiltration
Source: Pediatr Discov. 2023 Jun 9;1(1):e1. doi: 10.1002/pdi3.1 (PMC12118268; doi:10.1002/pdi3.1)
Supplement: Supplementary file 1 — Table S1 [file PDI3-1-e1-s001.docx]

***Table S1****. RNAseq Power*

| compare | depth | coefficient | sample  number | effect | FDR | RNASeq  power |
| --- | --- | --- | --- | --- | --- | --- |
| NEC_SC-VS-NOR | 16.727015 | 0.4 | 3,5 | 2 | 0.05 | 0.527514 |
| NEC-VS-NEC_SC | 17.058683 | 0.4 | 4,3 | 2 | 0.05 | 0.492069 |
| NEC-VS-NOR | 19.037031 | 0.4 | 4,5 | 2 | 0.05 | 0.610586 |
